# Assessing muscle spasticity with Myotonometric and passive stretch measurements: validity of the Myotonometer

**DOI:** 10.1038/srep44022

**Published:** 2017-03-10

**Authors:** Xiaoyan Li, Henry Shin, Sheng Li, Ping Zhou

**Affiliations:** 1 Department of Physical Medicine and Rehabilitation, University of Texas Health Science Center at Houston, and TIRR Memorial Hermann Research Center, Houston, TX, USA

## Abstract

Spasticity of the biceps brachii muscle was assessed using the modified Ashworth Scale (MAS), Myotonometry and repeated passive stretch techniques, respectively. Fourteen subjects with chronic hemiplegia participated in the study. Spasticity was quantified by muscle displacements and compliance from the Myotonometer measurements and resistive torques from the repeated passive stretch at velocities of 5 °/s and 100 °/s, respectively. Paired t-tests indicated a significant decrease of muscle displacement and compliance in the spastic muscles as compared to the contralateral side (muscle displacement: spastic: 4.84 ± 0.33 mm, contralateral: 6.02 ± 0.49 mm, p = 0.038; compliance: spastic: 1.79 ± 0.12 mm/N, contralateral: 2.21 ± 0.18 mm/kg, p = 0.048). In addition, passive stretch tests indicated a significant increase of total torque at the velocity of 100 °/s compared with that of 5 °/s (T_t5_ = 2.82 ± 0.41 Nm, T_t100_ = 6.28 ± 1.01 Nm, p < 0.001). Correlation analysis revealed significant negative relationships between the stretch test and the Myotonometer measurements (r < −0.5, p < 0.05). Findings of this study provided validation of the Myotonometry technique and its high sensitivity in examination of spasticity in stroke.

Spasticity is one of the most distinctive syndromes of upper motor neuron disorder, described as an abnormal resistance to external imposed movement^1^,^2^,^3^. Despite disagreements on the contributing mechanisms of spasticity, it is probably associated with increased reflex excitability, altered mechanical properties of passive tissues, or abnormal intrinsic properties of contractile elements^3^,^4^,^5^.

Characterization and quantification of spasticity remain essential for tracking disease progression or evaluating therapeutic interventions in rehabilitation^6^,^7^,^8^. Tendon reflex, Tardieu scale, and Modified Ashworth Scale (MAS) tests are frequently used in clinics for diagnosis of spasticity. In particular, the MAS test has been the standard for many newly-developed devices to be compared with^3^,^6^ due to its convenience in the clinical setting. On the other hand, the MAS test is also criticized as subjective and a tendency to cluster in the middle or lower ranges^9^. In addition, the differences between the grades are not quantitatively equal in the MAS because of the ordinal nature of the method^10^. As a result, validation of the MAS shows conflicting results of interrater reliability^9^,^11^,^12^.

Myotonometry is a new technique that provides objective assessment of muscle spasticity by quantifying tissue displacement with respect to perpendicular compression force. Other applications of the technique involve examination of the viscoelastic properties and compliance changes of muscle in subjects with stiff shoulders or scoliosis^10^,^13^,^14^,^15^. Reliability of the Myotonometry technique has been assessed by the intra- and interrater correlations or inter-session correlations in multiple muscles or different muscle conditions^16^,^17^,^18^,^19^. Myotonometer measurements have also been compared to conventional measurements such as the MAS, surface electromyography (EMG), and muscle stiffness from the stretch techniques^6^,^12^,^20^.

The stretch technique measures the changes in resistance torque during repeated joint rotations controlled by a servomotor^3^,^5^,^12^,^21^. By stretching the joint at a constant velocity while muscles were relaxed or voluntarily contracted with the delivery of electrical stimulations, it is possible to identify both the intrinsic and reflex components from the mechanical responses^12^,^22^. The technique has been used to quantify spasticity and explore its related pathophysiological mechanisms. Currently there is only one study that validates the Myotonometer measurement with the outcomes of the stretch technique in stroke and the study mainly evaluates the lower limb muscles^12^. Since subjects with spasticity also demonstrate contractures in heel cords (Achilles tendon)^3^,^23^, it remains unclear whether the contractures influence measurement of stiffness in calf muscles.

This study focuses on validation of the Myotonometry by comparing the measurement with the conventional stretch technique and the MAS test. Different from the previous study which involves sinusoidal stretches in small perturbations^12^, this study utilized a passive ramp-and-hold protocol with a much wider range to simulate typical clinical tests of spasticity. In addition, a two-layer spring model^24^ was applied to identify the muscle displacement from the overall tissue displacements in Myotonometer analysis. Findings of the study included a significant decrease of muscle compliance and displacement in Myotonometer measurements and a substantial increase of total torque in the high-speed passive stretch in the spastic side. Additionally, significant negative correlations were observed between the two measurements, which may provide evidence of validity of Myotonometry in the upper limb muscles.

## Results

The MAS test was performed in all subjects. To summarize, there was one score of 3, two scores of 2 and the rest of MAS scores were 1+ or below. The averaged months since stroke were 61 ± 30 months (mean ± std).

### Muscle compliance

**Figure 1:**
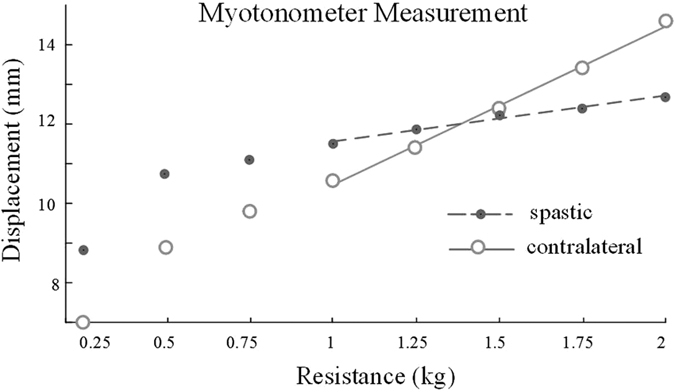
Tissue displacements sampled at different resistance from 0.25 kg to 2 kg at 0.25 kg increments in individual trials from the spastic and contralateral muscles. Spastic: dots and dash line; contralateral: open circle and solid line. Area under the curve for muscle (AUC_muscle) and muscle compliance were calculated within resistance from 1 kg to 2 kg. Muscle compliance was estimated from the slope of the lines.

An example of tissue displacement as a function of resistance is illustrated in Fig. 1 from a representative subject. Calculation of the area under the curve for the muscle (AUC_muscle) indicated a distinct difference between the spastic and contralateral sides (spastic: 3.2 mm, contralateral: 9.3 mm). In addition, muscle displacement showed strong linearity with the resistance in both sides (spastic: r^2^ = 0.99, p < 0.001; contralateral: r^2^ = 0.99, p < 0.001). Muscle compliance obtained from the slope of the linear relationship was remarkably lower in the spastic muscle than that of the contralateral side for the individual (spastic: 1.16 mm/kg; contralateral: 4 mm/kg).

**Figure 2:**
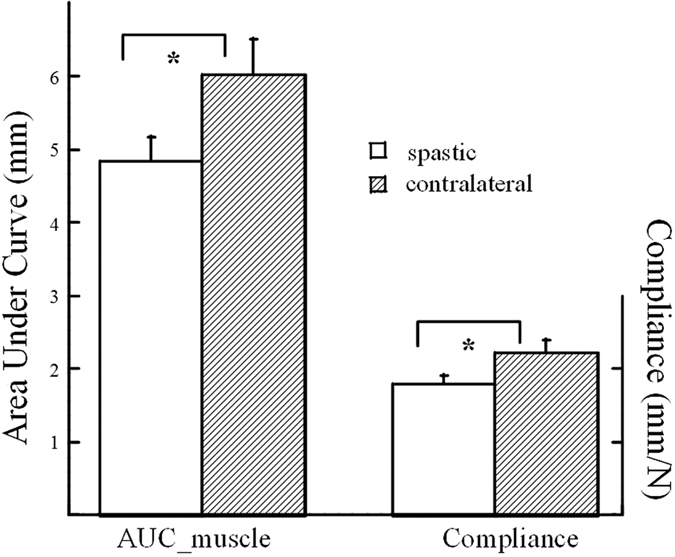
Comparisons of Myotonometric measurement between the spastic and contralateral muscles. Left: AUC_muscle; right: muscle compliance.

Regression analysis confirmed a significant linear relation between muscle displacement and resistance within 1 kg to 2 kg across all other subjects (spastic: r^2^ = 0.99, p < 0.001; contralateral: r^2^ = 0.98, p < 0.002). Comparison of the averaged AUC_muscle and muscle compliance from all subjects revealed a significant reduction of values in the spastic muscles compared with the contralateral side (Fig. 2. AUC_muscle: spastic: 4.84 ± 0.33 mm, contralateral: 6.02 ± 0.49 mm, p = 0.038. Muscle compliance: spastic: 1.79 ± 0.12 mm/N, contralateral: 2.21 ± 0.18 mm/kg, p = 0.048).

### Stretch test

**Figure 3:**
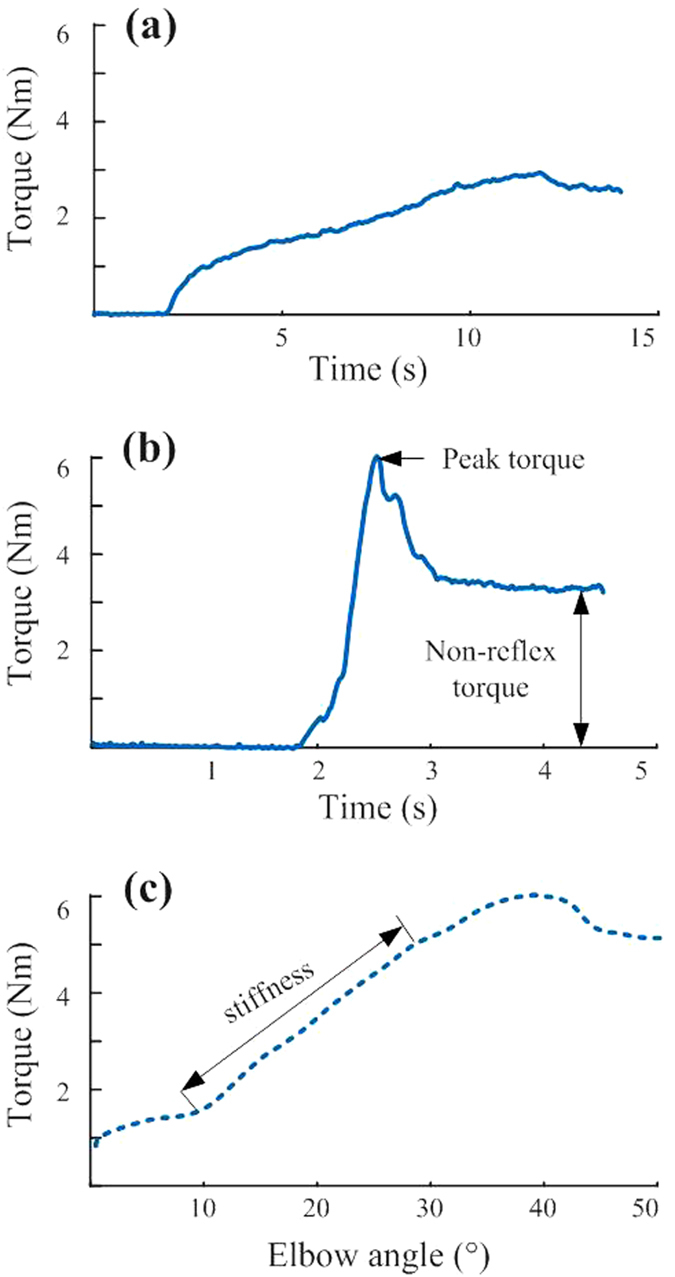
Elbow flexor torque collected from the same subject as Fig. 1. (**a**) Stretch speed: 5 °/s, (**b**) Stretch speed: 100 °/s. (**c**) The relation of torque-elbow angular displacement at 100 °/s, from the same trial as (**b**).

Examples of the elbow flexor torque at velocities of 5 °/s and 100 °/s are illustrated in Fig. 3 (3a and 3b) from the same subject in Fig. 1. The total torque at 5 °/s was substantially lower compared with that at 100 °/s (T_t5_ = 3.93 Nm, T_t100_ = 6.01 Nm). Reflex torque (2.08 Nm) was calculated as the difference of the total torques between the two speeds. The non-reflex torques at 5 °/s and 100 °/s are 2.58 Nm and 3.21 Nm, respectively. The function of resistive torque with elbow angular displacement at a speed of 100 °/s is plotted in Fig. 3c, where muscle total stiffness (0.17 Nm/°) was estimated from the linear segment of the plot.

**Figure 4:**
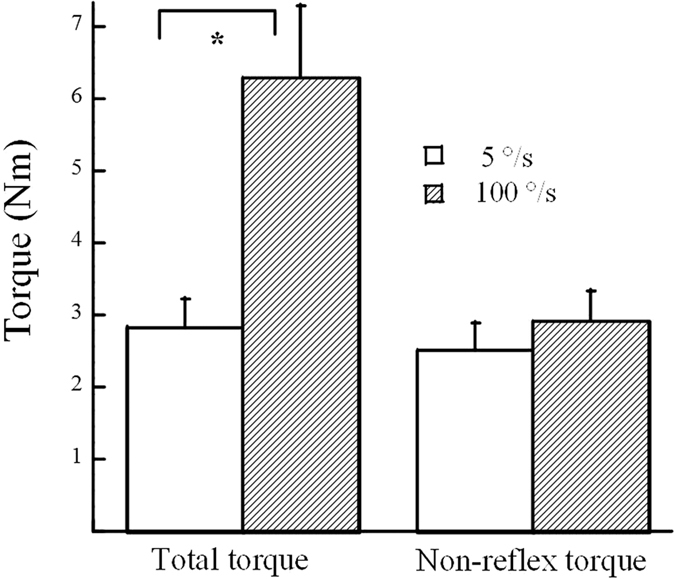
Comparisons of torques between 5 °/s and 100 °/s. Left: total torque; right: non-reflex torque.

Total torques averaged over all subjects were compared between two different velocities. A significant increase of torque was observed at the velocity of 100 °/s (T_t5_ = 2.82 ± 0.41 Nm, T_t100_ = 6.28 ± 1.01 Nm, p < 0.001, Fig. 4). Comparison of the non-reflex torque between the two speeds, however, did not show any significant changes (T_nr5_ = 2.51 ± 0.37 Nm, T_nr100_ = 2.92 ± 0.42 Nm, p = 0.13). As a result, the non-reflex torques from two speeds were pooled and the averaged values were used for correlation analysis.

### Correlations analysis

Correlation coefficients between the Myotonometric and stretch measurements were calculated and presented in Table 1. A significant negative relationship was observed between the total stiffness (from the stretch test at 100 °/s) and the muscle compliance variables (AUC_muscle and compliance from the Myotonometer test). No other associations were found between the Myotonometric and stretch measurements (p > 0.09).

Evaluation of the associations between the clinical assessments (months since stroke or the MAS) and the Myotonometric or stretch measurements did not confirm any correlations by calculating the Pearson coefficients or the Spearman ρ coefficients.

## Discussion

This study examined muscle spasticity in chronic stroke survivors using Myotonometry and conventional passive stretch techniques. Both techniques identified substantial changes in the spastic muscles, which included significantly reduced muscle compliance and displacements in the Myotonometric measurement and a substantial increase of total torque in the high-speed passive stretch test. Correlation analysis indicated a significant linear relation between the two measurements.

### Correlations between Myotonometric and stretch measurements

Myotonometry and conventional stretch techniques represent two different types of measurements. Muscle compliance obtained from the Myotonometer reflects the degree of muscle deformation with respect to the compression applied perpendicularly to the muscle^10^,^25^. Thus, it characterizes the viscoelastic properties of individual muscles, particularly the superficial ones. Muscle tonic contraction is one of the primary contributors to changes of muscle compliance^6^,^25^, which correlates well with different activation levels and strength^6^,^20^,^24^,^26^,^27^. Other physiological parameters that are associated with muscle compliance include composition of muscle fiber types, fascicle length, pennation angle, etc.^15^,^28^,^29^. Such parameters are altered in the progression of muscle atrophy, denervation and reinnervation after a stroke. In contrast, muscle stiffness reflects the joint resistance torque with respect to angular deflection and is typically measured in a dynamic process of passive elbow rotation. According to the literature, the total stiffness consists of the reflex, intrinsic, and passive components, which are associated with motoneuron discharge and excitability, intrinsic properties of the contractile apparatus, or mechanical (viscoelastic) properties of the passive tissues respectively^3^,^4^,^5^,^30^. The correlations of the two different techniques observed in our study may suggest that both techniques are capable of detecting muscle physiological changes associated with spasticity.

A significant negative linear relation between the intrinsic mechanical stiffness and muscle compliance was reported in the previous study, which suggested that the reduced compliance in the spastic muscle might reflect the alterations of the contractile properties of the muscle^12^. The passive stiffness and intrinsic stiffness, however, were not distinguished in the present study and were generally described as the non-reflex components. Examination of the non-reflex components with Myotonometer measurements did not reveal any significant correlations in our study. There are a number of factors leading to the different findings of our study and the previous one. For example, different stretch protocols and muscles are involved in the two studies. In addition, different data processing methods were used for Myotonometer analysis. In the previous study muscle compliance was characterized as the sum of tissue displacements from all resistance levels which did not distinguish the muscle displacement and subcutaneous tissue displacement^12^.

### Association between the MAS and biomechanical measures

MAS is the primary clinical assessment of spasticity that evaluates muscle resistance to passive movement, which assumes : 1) changes of resistance are exclusively due to spasticity; and 2) stretching velocity as well as range of movement (except grade 4) remain unchanged in the repeated measures^9^. On the other hand, resistance and range of movement are often affected by the level of muscle activity, the viscoelastic properties of the joint, the temperature, etc^3^,^31^,^32^,^33^. Therefore, validity of the MAS with other clinical assessments or biomechanical techniques has showed conflicting results and poor reliability^9^,^33^,^34^,^35^.

The MAS score was not correlated with the Myotonometric measurements in this study whereas other groups found significant correlations^6^,^12^. The differences may be partly due to the less reliability of the MAS and the different muscle activities quantified. In particular, muscle displacements were quantified in both relaxation and voluntary contractions in previous studies and were evaluated only in the relaxation in the current study. A lack of significant correlation was also observed between the MAS and the passive stretch stiffness in this study. In the literature, examination of such correlations in the lower limb revealed controversial findings using similar techniques^12^,^33^. The insignificant correlations in our study could be associated with the relative small range of MAS scores, most of which are within grades ‘1’ and ‘1+’. Given the relative large variations in the torque and Myotonometric measurements in stroke and the tendency of MAS clustering in the lower range, this may interpret the insignificant relations between the MAS and the biomechanical measures in our study.

### Limitations

As tonic muscle contraction has a substantial influence on muscle compliance^10^,^25^, current assessment of muscle compliance from only the relaxed condition may limit the generalizability of our findings. Future studies examining muscle compliance at different contraction levels may provide new information on the validity of the Myotonometry technique. In the present study, a standardized elbow position (90 degrees of elbow flexion) was used. This procedure was advantageous to minimize measurement errors for Myotonometer and muscle stretch tests. However, it is a limitation when muscle compliance and stiffness were correlated with clinical assessment (MAS scores). It is known that high correlations between reflex torque and stiffness and MAS scores were observed when stretch tests were applied with reference to their preferred resting joint position^36^. No such correlations were observed when a standardized elbow joint position was selected for subjects with various levels of spasticity, i.e., MAS scores^33^.

## Conclusion

This study identified significant changes in the spastic muscles using the Myotonometry and conventional stretch techniques. The significant correlations between the two measurements indicated high sensitivity of the Myotonometry technique to the detection of spasticity and provided a validation of the technique.

## Methods

### Subjects

Fourteen subjects (8 F 6 M, aged 61 ± 10 years, mean ± std) with chronic hemiplegia were recruited for the study. They only had a single incidence of stroke and were free of any other known neurological disorders. The time course of stroke ranged from 6 months to 8 years and 5 months. Experimental protocols and the informed consent were approved by the Institutional Review Board of University of Texas Health Science Center and TIRR Memorial Herman (Houston, USA). Informed consent was obtained from all subjects prior to the experiments. In addition, all methods were performed in accordance with relevant guidelines and regulations. The clinical assessment included the Modified Ashworth Scale (MAS) test on the spastic elbow flexors.

### Experiments

#### Muscle compliance test

Subjects were seated comfortably in a height-adjustable chair having the shoulder slightly abducted and the elbow flexed in 90 °. A Myotonometer (Neurogenic Technologies, Missoula, MT) was used to measure the tissue displacement and corresponding resistance against the tissue deformation. Prior to a trial, the Myotonometer was positioned on the bulk of the biceps brachii muscle perpendicularly to the skin. The two components of the sensor, the inner probe and outer sleeve, positioned at the same level on the surface with no force applied to the Myotonometer. During the test, subjects were instructed to remain relaxed as the experimenter pressed the handle of the Myotonometer downward into the muscle. As more compression was applied on the handle, the depth of probe penetration increased whereas the outer sleeve remained static. Thus, tissue displacement was measured as the distance between the probe and the plastic sleeve. The Myotonometer recorded both tissue displacement and resistance simultaneously. In particular, the tissue displacement was sampled at eight resistance levels from 0.25 kg to 2.0 kg at increments of 0.25 kg. The test was performed bilaterally for eight trials on each of the biceps brachii muscles for all subjects.

#### Muscle stretch test

Subsequent to the compliance test, subjects took a brief rest before participating in the stretch test. Their spastic arm was fastened in a customized manipulandum with the shoulder in 45 °of abduction and 30 ° of flexion. In addition, the elbow joint was aligned to the vertical axis of the torque sensor (Model TRS 500, Transducers Techniques, CA) where a servomotor (HD FHA-25C-50-US250, Harmonic Drive LLC, MA) was mounted and aligned. Such configuration was used to minimize translational and rotational movements of the arm during the stretch.

Subjects were instructed to relax while their arm was passively stretched by the servomotor. A ramp-and-hold protocol was applied to all subjects in the study. The sequence of the protocol consisted of a rest period of 2 s, a constant velocity stretch of elbow flexors, a 2 s holding pause, and a return to the initial position at the same constant velocity. The total range of stretch was 50 ° and the initial position was determined by each individual’s resting angle. After the stretch, a brief rest of 30 seconds was given to provide sufficient recovery time and minimize the effect of stretch on the muscle in the next trial. The stretch was performed at two different velocities of 5 °/s or 100 °/s respectively. Each velocity was tested in three trials.

The elbow flexor torque and angular position were recorded and digitized to the computer via a data acquisition board (BNC-2090 A, National instruments, TX) at a sampling frequency of 1000 Hz.

### Data analysis

Offline analysis of the Myotonometric and stretch parameters was conducted in MATLAB^®^ (MathWorks, Natick, MA).

#### Muscle compliance analysis

As illustrated in Fig. 1, a remarkable change of tissue displacement occurs around 0.75 kg of resistance or lower in spastic and contralateral muscles. Such change was also observed in all other subjects. It is assumed that the larger tissue displacement appears in the more superficial tissues before the muscle is compressed^24^,^25^. Therefore, muscle displacement in this study was defined as the resistance varied from 1 kg to 2 kg. As a result, the area under the curve of the muscle was calculated as the sum of muscle displacement with respect to the displacement at 1 kg (AUC_muscle = 
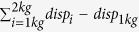
). Muscle compliance was estimated from the slope of significant linear relation between muscle displacement and resistance (the lines in Fig. 1). Since all muscle demonstrate significant linear relation between the displacement and resistance, AUC_muscle and compliance were calculated for all subjects in the spastic and contralateral sides.

### Stretch analysis

The elbow torque collected from the stretch-and-hold protocol was smoothed with low-pass filter of 8 Hz prior to analysis. Next, peak torque was searched within a time window between onset of stretch and two seconds after the completion of the stretch. Total torque (T_t_) was defined as the difference between peak torque and the baseline torque (or the resting torque) in the trial. According to the literature, stretch reflex is likely elicited by rotation of the elbow joint at high speeds^37^. Therefore, total torque at 100 °/s was assumed to represent the sum of stretch reflex-mediated response and the non-reflex response. It is also agreed that torque recorded at 6 °/s or lower is insensitive to reflex activity^30^. Thus, reflex torque (T_r_) of the elbow flexors was calculated as the difference of the total torque between 5 °/s and 100 °/s (T_r_ = T_t100_ - T_t5_) in the study. The non-reflex torque (T_nr_), which represents the sum of the passive and intrinsic components, was calculated as the torque averaged over a 1 s time window in the end of the holding period. Muscle total stiffness was estimated from the total torque-angular displacement relation in the selected linear segment. Examples of the elbow flexor torques at different speeds and examples of peak torque, no-reflex torque and muscle total stiffness were illustrated in Fig. 3.

### Statistical analysis

Paired t-test was applied to compare the differences of Myotonometric variables including the AUC_muscle and muscle compliance between the spastic and contralateral muscles. The same test was applied to compare the differences of total torque and the non-reflex torque between different speeds of 100 °/s and 5 °/s. Pearson correlation analysis was used to assess any linear relations between the Myotonometer variables and the stretch torques (reflex torque, non-reflex torque, and total torque and stiffness at 100 °/s). Similarly, Pearson correlation was applied to assess the linearity between the duration of the stroke and the Myotonometer variables or stretch torques. Due to the ordinal nature of the MAS, Spearman ρ coefficients were computed to examine whether the MAS was correlated with Myotonometer or stretch variables. All data were presented in the format of mean ± standard error unless specified. Statistical significance was defined as p <0.05.

## Additional Information

**How to cite this article:** Li, X. *et al*. Assessing muscle spasticity with Myotonometric and passive stretch measurements: validity of the Myotonometer. *Sci. Rep.* **7**, 44022; doi: 10.1038/srep44022 (2017).

**Publisher's note:** Springer Nature remains neutral with regard to jurisdictional claims in published maps and institutional affiliations.

## Figures and Tables

**Table 1:** Correlation coefficients of Myotonometric and stretch variables.

	AUC_Muscle	Compliance
Reflex Torque	−0.467 (0.092)	−0.438(0.117)
Non-reflex Torque	−0.468 (0.092)	−0.439 (0.116)
Total Torque (T_t100_)	−0.452 (0.105)	−0.411 (0.144)
Stretch stiffness	−**0.607 (0.021)**	**−0.556 (0.039)**

Significant coefficients are in bold. P values are in parentheses.

AUC_Muscle: area under the curve for muscle.
